# The blending region hybrid framework for the simulation of stochastic reaction–diffusion processes

**DOI:** 10.1098/rsif.2020.0563

**Published:** 2020-10-21

**Authors:** Christian A. Yates, Adam George, Armand Jordana, Cameron A. Smith, Andrew B. Duncan, Konstantinos C. Zygalakis

**Affiliations:** 1 Department of Mathematical Sciences, University of Bath, Claverton Down, Bath BA2 7AY, UK; 2 Centre de Mathématiques et de Leurs Applications, CNRS, ENS Paris-Saclay, Université Paris-Saclay, 94235 Cachan cedex, France; 3 Department of Mathematics, Imperial College London, London SW7 2AZ, UK; 4 School of Mathematics, University of Edinburgh, James Clerk Maxwell Building, The King’s Buildings, Peter Guthrie Tait Road, Edinburgh EH9 3FD, UK

**Keywords:** hybrid modelling, stochastic reaction–diffusion, multiscale modelling, partial differential equation, hybrid modelling framework

## Abstract

The simulation of stochastic reaction–diffusion systems using fine-grained representations can become computationally prohibitive when particle numbers become large. If particle numbers are sufficiently high then it may be possible to ignore stochastic fluctuations and use a more efficient coarse-grained simulation approach. Nevertheless, for multiscale systems which exhibit significant spatial variation in concentration, a coarse-grained approach may not be appropriate throughout the simulation domain. Such scenarios suggest a hybrid paradigm in which a computationally cheap, coarse-grained model is coupled to a more expensive, but more detailed fine-grained model, enabling the accurate simulation of the fine-scale dynamics at a reasonable computational cost. In this paper, in order to couple two representations of reaction–diffusion at distinct spatial scales, we allow them to overlap in a ‘blending region’. Both modelling paradigms provide a valid representation of the particle density in this region. From one end of the blending region to the other, control of the implementation of diffusion is passed from one modelling paradigm to another through the use of complementary ‘blending functions’ which scale up or down the contribution of each model to the overall diffusion. We establish the reliability of our novel hybrid paradigm by demonstrating its simulation on four exemplar reaction–diffusion scenarios.

## 1. Introduction

Many biological and physical systems are inherently multiscale in nature [1–6]. The modelling of such systems, therefore, requires multiscale representations which, by their nature, are not well captured using a single modelling paradigm. There is a trade-off between, on the one hand, ensuring that models are sufficiently detailed that they accurately capture known biological and physical phenomena of interest and, on the other, achieving model outputs in a timely manner.

The appropriate representation of travelling waves of cells in developmental or maintenance contexts is a classic example of a multiscale phenomenon for which the trade-off between cheap-but-coarse and expensive-but-accurate modelling paradigms is evident. For a pulled wavefront, the wave speed is determined by the low-density dynamics at the front of the wave [7]. It is, therefore, important to represent cell movement and proliferation dynamics at the front using an appropriately detailed model. A model that is too coarse may neglect important features of the real process. Behind the wave, cell density is higher, making a fine-grained representation more computationally expensive. Since the fine details are less important in this region, we can substitute the more detailed model for a cheaper, coarser representation. Coupling modelling regimes at different scales is an open question to which a variety of solutions have previously been proposed [1,8–33]. For more details on the different types of hybrid methods available, we direct the interested reader to Smith & Yates [34].

In this paper, we focus on the three main modelling paradigms used for representing reaction–diffusion systems. At the coarsest scale (which we refer to as the *macroscopic* scale), we represent the *concentration* of reactant species by partial differential equations (PDEs) [35–41]. For validity, these models typically require high concentrations since assumptions underlying the use of PDEs break down for low copy numbers. Continuum models such as these can usually be simulated extremely efficiently using a wide variety of well-established numerical methods; however, they lack the realism of finer-scale models.

At the next level down, the *mesoscopic* scale, reactant species are represented as individual particles and are compartmentalized into contiguous, non-overlapping subdivisions of space [4,42–48]. Particles are assumed to be well-mixed within a compartment and can interact with others in their compartment. These models can capture stochasticity in the behaviour of the particles and can be simulated efficiently when copy numbers are low. However, when particle numbers become large, simulations can become prohibitively slow in comparison to macroscale representations. They also lack the accuracy of more fine-grained models since the individual particle identities and positions are not retained.

The finest representation we consider is a Brownian dynamics model at the *microscopic* scale [49–52]. In these models, the trajectories of all particles are simulated (typically using a discrete fixed time-step paradigm) in continuous space [49,53–55]. For a system of *N* particles, an appropriate simulation algorithm must generate *ZN* Gaussian random variables (where *Z* is the dimension of the system) in order to update the particle positions. For simulations incorporating pairwise interactions, *N*^2^ pairwise distances must also be updated at each time step.^1^ Consequently, these methods can be extremely computationally intensive. They do, however, provide a comprehensive and accurate individual representation capable of incorporating stochasticity into particle positions and interaction times. More details on the specific implementation of each of these three modelling paradigms will be given in the next section.

In general, the aim of a hybrid method is to exploit the complementary advantages and negate the complementary weaknesses of models at different scales. Using a coarse, cheap representation in a region of space in which particle density is high allows for significant computational savings in comparison to the purely fine-scale simulation. Conversely, implementing a fine-scale individual-based representation in regions in which low-copy-number effects are of paramount importance can give significant improvements in accuracy in comparison to coarser models. Consequently, one way to achieve accurate simulations that are also computationally tractable is to combine the models’ strengths in a hybrid representation.

In this paper, we propose a novel hybrid method for coupling PDEs at the macroscale to compartment-based models at the mesoscale and a related novel hybrid method for coupling compartment-based models at the mesoscale to Brownian dynamics models at the microscale. In each case, the coarser regime is coupled to the finer regime through an overlap region. In this overlap region, which from now on we will refer to as the *blending region*, both representations of the reaction–diffusion dynamics are valid. In the blending region, the strength of diffusion for each model is determined by a spatially varying *blending function*, which is prescribed to be unity on one end of the overlap region and zero on the other. The blending functions for the two models are complementary so that the sum of the two blending functions at any point in the domain is equal to unity. These functions control the relative contribution of each model to the diffusion dynamics. This approach is reminiscent of that taken by Duncan *et al.* [57] in a non-spatial context. In Duncan *et al.* [57], two different non-spatial models for stochastic chemical kinetics were coupled in copy-number space through a blending region in which both models coexisted.

The remainder of the paper is organized as follows. In §2, we describe the individual reaction–diffusion models that we couple together and provide a brief justification for why the models can be considered ‘equivalent’ and hence are suitable candidates for coupling. In §3, we present the mechanics of the two-hybrid blending methods and prove their effectiveness, in §4, by simulating a number of test scenarios and determining whether any bias is introduced by the blending methods. We conclude in §5 with a short summary of our findings and suggestions for extensions to this work.

## 2. Modelling at different scales

Within this section, we describe the three different modelling scales that we will couple in order to create two distinct spatially coupled hybrid methods. In §2.1, we describe a general macroscale PDE for reaction–diffusion systems with a single species, as well as different numerical approaches for its solution. Section 2.2 contains a discussion of mesoscale compartment-based models and their simulation, while in §2.3 we introduce the microscale individual-based dynamics. In §2.4, we briefly discuss how each of these representations of reaction–diffusion processes at different scales might be considered to be equivalent in an appropriate limit.

### 2.1. Macroscopic representation

Partial differential equations, the macroscale models we employ in this paper, can be considered to be appropriate representations of the mean behaviour of particles at high concentrations. The primary advantage of the PDE representation is that there exists a wide range of well-established and well-understood tools for their numerical simulation. In rare, simple cases, PDEs are amenable to mathematical analysis. However, they typically fail to model low-copy-number behaviour.

A generic PDE which describes the spatio-temporal evolution of the concentration of a single species, *c*(***x***, *t*), at position ***x*** and time *t* takes the form

2.1 $$\frac{\partial c}{\partial t}(x,t)=\nabla \cdot (D(x)\nabla c(x,t))+R(c(x,t),x,t),x\in R^{Z},\;t\in [0,T],$$

where consistent initial and boundary conditions need also to be specified. Here, reactions are represented by the function R, *Z* is the dimension of space and *T* is the final time to which we wish to evolve the solution. Note that the spatially varying diffusion coefficient, represented by *D*(***x***), sits inside the first derivative, but not the second. As noted by van Kampen [58], there is no canonical choice of operator describing spatially dependent diffusion. In physical applications, the form of the macroscopic diffusion equation should be dictated by the underlying microscopic or mesoscopic process. Since the spatial dependence of the diffusion coefficient in our hybrid methods is introduced purely as a modelling convenience and does not correspond to any microscopic or mesoscopic ground truth, we are effectively free to choose the form of the diffusion operator. We adopt the form considered by Benson *et al.* [59] (see equation (2.1)). We choose the transition rates in the corresponding compartment-based representation (see §2.2) and the drift and diffusion coefficients of the corresponding microscopic position evolution equation (see §2.3) so that diffusion in the overlap regions of the hybrid methods satisfies the same form of non-constant coefficient diffusion equation.

For the majority of this paper, we focus on the following one-dimensional PDE in the region *Ω* = [*a*, *b*]:

2.2 $$\frac{\partial c}{\partial t}=\frac{\partial }{\partial x}\left(D(x)\frac{\partial c}{\partial x}\right)+R(c(x,t)),$$

with constant flux boundary conditions

2.3 $$D(a)\frac{\partial c}{\partial x}{|}_{x=a}=J_{a},\;D(b)\frac{\partial c}{\partial x}{|}_{x=b}=J_{b}.$$

For a discussion of the implementation of the numerical solution of the PDEs employed in this paper please refer to appendix A. Note that there is no explicit spatial dependence in the reaction term in equation (2.2).

### 2.2. Compartment-based representation

Compartment-based methods are coarse-grained stochastic representations. The spatial domain is typically divided into compartments, each of size *h*, in which particles are assumed to be well-mixed. The reaction–diffusion dynamics are characterized by a set of possible events. Events are either reactions, in which particles can interact with others within their own compartment according to some prespecified reaction rates, or jumps to adjacent compartments with rates which depend on the macroscopic diffusion coefficient, *D*(*x*), and the compartment size, *h*. Specifically, in order to capture diffusion which corresponds to the macroscopic equation (2.1) we must choose the rates of jumping to be different depending on the direction of the jump (see equations (B 1) and (B 2) for more detail).

Throughout this paper, we refer to models at this scale as *mesoscopic* or *compartment-based*. For a discussion of the implementation of the numerical simulation of the compartment-based models employed in this paper please refer to appendix B.

### 2.3. Brownian-based representation

Individual-based methods require the recording and updating of large numbers of particles’ positions. Relative positions for each pair of particles must also be maintained at every step if higher-order reactions (higher than first order) or volume exclusion are to be modelled. For large particle numbers, *N*, the *O*(*N*^2^) computational complexity means that individual-based simulation algorithms can become extremely expensive.^2^

In what follows we employ a fixed-time-step algorithm, although we note that continuous-time algorithms for Brownian reaction–diffusion dynamics are also available [55]. The evolution of particle *i*’s position, *y*_*i*_(*t*), between times *t* and *t* + Δ*t*_*b*_ in the case of space-dependent diffusion (corresponding to PDE (2.2) and compartment-based jump-rates given by equations (B1) and (B2)) can be simulated according to the following discrete-time update equation:

2.4 $$\begin{array}{cc} y_{i}(t+\Delta t_{b}) & =y_{i}(t)+\Delta t_{b}\frac{dD(x)}{dx}{|}_{x=y_{i}(t)}\;\\ \; & \;+\sqrt{2D(y_{i}(t))\Delta t_{b}}\xi_{i},\; \end{array}$$

where *ξ*_*i*_ ∼ *N*(0, 1) is a Gaussian random variable with mean 0 and variance 1. If required, reactions can be implemented according to a variety of different algorithms [53,55]. In this paper, we employ the λ−ρ method [51]. If two eligible particles come within a reaction radius, *ρ*, of each other they interact with a given rate, *λ*, according to the appropriate reaction pathway.

We refer to these models at this scale as *off-lattice*, *microscopic* or *individual-based* models in what follows.

### 2.4. Connections between models at different scales

In attempting to couple together representations of the same phenomenon at different scales we need to ensure that, under certain assumptions, they are representations of the same process. Pioneering work in establishing the connection between stochastic and deterministic models was undertaken by [60–62]. In this section, we concisely summarize the ways in which the models outlined above can be considered to be equivalent and direct the interested reader to resources which contain more detailed arguments.

In order to transition from the mesoscale to the macroscale, we can first use the reaction–diffusion master equation to derive the deterministic mean-field representation of the compartment-based particle numbers [42,51,63]. It should be noted that for second- and higher-order reactions, the mean-field equations are only approximations of the true mean behaviour of the stochastic system [64]. Taking the diffusive limit of the mean-field equations gives a corresponding reaction–diffusion PDE.

The Fokker–Planck equation can be used to connect a microscale stochastic differential equation (SDE) model of diffusion to a macroscale model describing the evolution of the probability density of a particle’s position [64,65]. For example, the canonical diffusion equation is the macroscopic Fokker–Planck equation corresponding to non-interacting particles undergoing simple Brownian motion.

Although we do not use this macroscopic–microscopic coupling directly in this work, we employ it indirectly in order to link the microscopic and mesoscopic descriptions together through their connection to the same PDE. Alternatively, first-passage time theory can be applied to a particle which moves subject to a given SDE in order to derive jump rates between neighbouring compartments in a compartment-based representation [47,66]. Connections between the models at microscale and mesoscale are stated more rigorously by Isaacson [67].

## 3. Hybrid blending algorithms

In this section, we discuss the two main algorithms of this paper. In particular, in §3.1, we present the central unifying idea behind both of our hybrid methods. The methods can both be understood as operator-splitting algorithms in which, in a central overlap region between the two regimes, diffusion is dealt with by both regimes using spatially varying diffusion coefficients. We discuss how to couple the methods discussed in §2 in order to accommodate this split-diffusion paradigm. In §3.2, we give the specific details of how to convert mass from one modelling regime to another to ensure both models are synchronized and valid representations of the particle density in the blending region. We then present, in §3.3, a generic algorithm for coupling the PDE with the compartment-based approach, as well as a similarly general algorithm for coupling the compartment-based approach with Brownian dynamics. We emphasize that the generic methods we present for coupling two regimes are independent of the numerical implementations chosen to simulate each regime. However, for ease of use and reproducibility, we have provided details of the numerical implementations we chose in appendices A–C.

### 3.1. Hybrid modelling interpreted as a splitting algorithm

In order to illustrate the conceptual framework behind our algorithms, we consider the following constant coefficient diffusion PDE in *Ω* = [*a*, *b*]:

3.1 $$\frac{\partial c}{\partial t}=\frac{\partial }{\partial x}\left(D\frac{\partial c}{\partial x}\right),$$

with the following zero-flux boundary conditions:

3.2 $$D\frac{\partial c}{\partial x}{|}_{x=a}=D\frac{\partial c}{\partial x}{|}_{x=b}=0.$$

Divide the domain, *Ω*, into three subdomains *Ω*_1_ = [*a*, *I*_1_], *Ω*_2_ = [*I*_1_, *I*_2_], *Ω*_3_ = [*I*_2_, *b*] and write the constant diffusion coefficient *D* = *D*_1_(*x*) + *D*_2_(*x*), where

3.3 $$D_{1}(x)=\left\{ \begin{array}{cc} D,\; & a\leq x<I_{1},\;\\ \;f_{1}(x),\; & I_{1}\leq x<I_{2},\;\\ 0,\; & I_{2}\leq x\leq b\; \end{array}\right.$$

and

3.4 $$D_{2}(x)=\left\{ \begin{array}{cc} 0,\; & a\leq x<I_{1},\;\\ \;f_{2}(x),\; & I_{1}\leq x<I_{2},\;\\ D,\; & I_{2}\leq x\leq b,\; \end{array}\right.$$

where *f*_1_ and *f*_2_ are monotonically decreasing/increasing functions, respectively, with *f*_1_(*x*) = *D* − *f*_2_(*x*) and *f*_1_(*I*_1_) = *f*_2_(*I*_2_) = *D* and *f*_1_(*I*_2_) = *f*_2_(*I*_1_) = 0 in order to ensure continuity of *D*_1_ and *D*_2_ across *Ω*.

Equation (3.1) can now be written as

3.5 $$\frac{\partial c}{\partial t}=\underset{1}{\underbrace{\frac{\partial }{\partial x}\left(D_{1}(x)\frac{\partial c}{\partial x}\right)}}+\underset{2}{\underbrace{\frac{\partial }{\partial x}\left(D_{2}(x)\frac{\partial c}{\partial x}\right)}},$$

with corresponding boundary conditions

3.6 $$(D_{1}(x)+D_{2}(x))\frac{\partial c}{\partial x}{|}_{x=a}=D\frac{\partial c}{\partial x}{|}_{x=a}=0\;\text{and}\;(D_{1}(x)+D_{2}(x))\frac{\partial c}{\partial x}{|}_{x=b}=D\frac{\partial c}{\partial x}{|}_{x=b}=0.$$

In addition, we specify the initial condition *c*(*x*, 0) = *c*_0_(*x*). It is straightforward to show that, because *D*_1_(*x*) = 0 in [*I*_2_, *b*], the operator indicated by 1 in equation (3.5) does not influence the concentration of *c* in that region. In a similar way, because *D*_2_(*x*) = 0 in [*a*, *I*_1_], the operator indicated by 2 in equation (3.5) does not influence the concentration of *c* in that region. Now let $\phi_{\tau }^{\left(1\right)},\phi_{\tau }^{\left(2\right)}$ be the flow maps associated with the propagation of the operators 1 and 2 in equation (3.5) until time *τ*. Specifically, this means that the solution of the following equations:

3.7a $$\frac{\partial c^{\left(1\right)}}{\partial t}=\frac{\partial }{\partial x}\left(D_{1}(x)\frac{\partial c^{\left(1\right)}}{\partial x}\right),\;D_{1}(a)\frac{\partial c^{\left(1\right)}}{\partial x}{|}_{x=a}=D_{1}(I_{2})\frac{\partial c^{\left(1\right)}}{\partial x}{|}_{x=I_{2}}=0$$

and

3.7b $$\frac{\partial c^{\left(2\right)}}{\partial t}=\frac{\partial }{\partial x}\left(D_{2}(x)\frac{\partial c^{\left(2\right)}}{\partial x}\right),\;D_{2}(I_{1})\frac{\partial c^{\left(2\right)}}{\partial x}{|}_{x=I_{1}}=D_{2}(b)\frac{\partial c^{\left(2\right)}}{\partial x}{|}_{x=b}=0,$$

subject to initial conditions $c^{\left(i\right)}(x,0)=c_{0}^{\left(i\right)}(x)$, can be written as $c^{\left(i\right)}(x,\tau )=\phi_{\tau }^{\left(i\right)}(c_{0}^{\left(i\right)})(x),\;\text{for}\;i=1,2$, respectively.^3^

The idea behind splitting methods is that one can now obtain an approximation for the solution of equation (3.5) at time *τ* by using an appropriate composition of the flow maps $\phi_{\tau }^{\left(1\right)}$ and $\phi_{\tau }^{\left(2\right)}$. In particular, the simplest splitting method is given by

3.8 $$c(x,\tau )\approx (\phi_{\tau }^{\left(1\right)}\circ \phi_{\tau }^{\left(2\right)})(c_{0})(x),$$

where we note that the ordering of the composition is unimportant.

At first glance, this seems like an unnecessarily complicated approach for obtaining an approximation for the solution of equation (3.1). However, choosing the flow maps $\phi_{\tau }^{\left(1\right)}$ and $\phi_{\tau }^{\left(2\right)}$ to represent propagation operators for two different model types allows us to seamlessly blend the distinct numerical update rules of the different modelling regimes described in §2. For example, when coupling the PDE to the compartment-based model, $\phi_{\tau }^{\left(1\right)}$ might represent an update operator for the numerical solution of the PDE up to time *τ*, while $\phi_{\tau }^{\left(2\right)}$ might represent steps of the position-jump Markov processes described in §2.2 up until time *τ*.

**Figure 1.**
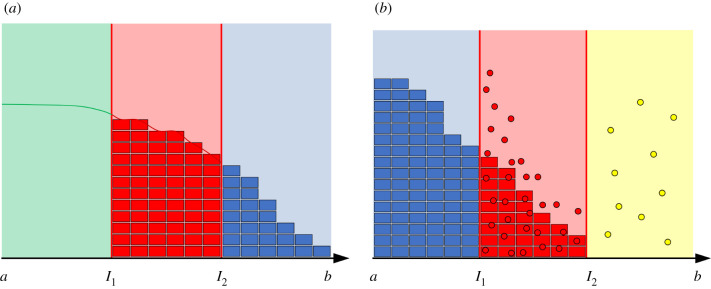
Schematic of (*a*) the PDE–compartment hybrid and (*b*) the compartment–Brownian hybrid. In (*a*), the green curve in the green region [*a*, *I*_1_] represents the PDE solution in the purely PDE region of the domain. The red curve and the red boxes represent equivalent PDE- and compartment-based representations of the mass in the red blending region. The blue boxes in the blue region of the domain represent the number of particles in each compartment in the purely compartment region of the domain. In (*b*), the blue boxes in the blue region of the domain represent the number of particles in each compartment in the purely compartment region of the domain. The red boxes and the red circles represent equivalent compartment- and Brownian-based representations of the mass in the red blending region. The yellow circles in the yellow region of the domain represent individual particles in the purely Brownian region of the domain. Note that we have given each Brownian particle a different height to aid clarity of visualization, but in reality all particles lie on the *x*-axis in these one-dimensional simulations.

Owing to the properties of the diffusion functions *D*_*i*_(*x*), the two models only coexist in the blending region [*I*_1_, *I*_2_]. Therefore, in applying the operator splitting update illustrated in equation (3.8), we only need to worry about how the concentration of the numerical solution of the PDE in the blending region translates to particle numbers for the compartment-based approach and vice versa. We must ensure that any PDE solution update in the blending region implemented by operator $\phi_{\tau }^{\left(1\right)}$ is also reflected in the compartment-based solution. Equivalently, any update to the compartment-based solution in the blending region implemented via $\phi_{\tau }^{\left(2\right)}$ must be reflected in the PDE solution. In a similar way, when coupling the compartment-based model to Brownian dynamics, one need only worry about how the particle numbers for the compartment-based approach in the blending region impact on the particle positions of the off-lattice Brownian dynamics and vice versa. Outside the two blending regimes, the two representations are effectively decoupled in terms of their update operators. In figure 1, we provide a schematic representation of (*a*) the PDE–compartment hybrid and (*b*) the compartment--Brownian hybrid for illustrative purposes.

### 3.2. Conversion rules

In this section, we illustrate how to couple two distinct representations of reaction–diffusion processes in the blending region. First, we tackle a PDE–compartment-based hybrid pairing, followed by a coupling between compartment-based and Brownian-based particle dynamics.

#### 3.2.1. Conversion between PDE and compartment-based model

We assume that the numerical solution of the PDE is calculated on the discrete mesh^4^ (see figure 7 in appendix A for an illustration) of size Δ*x* in [*a*, *I*_2_] and that compartment-based dynamics are simulated with compartments of size *h* in [*I*_1_, *b*]. It is natural to assume that *h* ≥ Δ*x*, as a fine discretization of the PDE mesh is required in order to minimize the error between the numerical solution and the exact solution it approximates. Note, however, that this is not a limitation of our algorithm and that *h* ≤ Δ*x* would also be possible. There are *n*_1_ = (*I*_2_ − *I*_1_)/Δ*x* PDE solution voxels in the overlap region [*I*_1_, *I*_2_] and *n*_2_ = (*I*_2_ − *I*_1_)/*h* compartments in the same region, where $n_{1},n_{2}\in N$. For ease of computation, we assume that *n*_1_ = *γn*_2_, with γ∈N so that there are an integer number of PDE solution voxels per compartment. There are also *n*_*p*_ = (*I*_1_ − *a*)/Δ*x* PDE solution voxels in the purely PDE region, [*a*, *I*_1_], and *n*_*c*_ = (*b* − *I*_2_)/*h* compartments in the purely compartment-based regime, [*I*_2_, *b*]. The numerical solution of the PDE in voxel *i* is labelled *q*_*i*_ for *i* = 1, …, *n*_*p*_ + *n*_1_ and the number of particles in compartment *i* is labelled *C*_*i*_ for *i* = 1, …, *n*_2_ + *n*_*c*_.

In each time interval of length Δ*t*_*p*_ we assume, without loss of generality, that the PDE solution is updated first and the compartment-based solution second. After the propagation of the discrete PDE solution operator in the time interval [*t*, *t* + Δ*t*_*p*_], assume that the concentrations in PDE voxels of the blending region have changed. Consequently, it is necessary to modify the corresponding compartment-based description in the blending region [*I*_1_, *I*_2_] before propagating the compartment-based model in the region [*I*_1_, *b*]. More precisely, for compartment *i* in the blending region, set

3.9 $$C_{i}=\sum_{\;j=1}^{\gamma }q_{n_{p}+\gamma (i-1)+j}\Delta x.$$

Because we are required to synchronize the representations of the solutions in the two regimes according to equation (3.9), the number of particles contained in the *i*-th compartment in the blending region is no longer an integer. Nevertheless, when it comes to performing the stochastic simulation algorithm, we work with these non-integer values to calculate the time until the next event. This could potentially be an issue when the copy numbers in a compartment are low, but arguably this would imply that we were using the PDE description to represent concentrations in a region of the domain for which this is not appropriate. A similar synchronization is implemented once the compartment-based model has been propagated and the number of particles in the blending region has changed. In particular, if *δC*_*i*_ corresponds to the integer change in particle numbers in the compartment *i* in the blending region, then one adds uniformly *δC*_*i*_/*γ*Δ*x* to the PDE solution in each of the PDE voxels, i.e.

3.10 $$q_{n_{p}+\gamma i+j}=q_{n_{p}+\gamma i+j}+\frac{\delta C_{i}}{\gamma \Delta x},\;j=1,\ldots ,\gamma .$$

Reactions in the blending region are always implemented according to the compartment-based paradigm. If reactions occur then particle numbers in compartments are updated and the corresponding change is also implemented in the appropriate PDE voxels, as in equation (3.10).

#### 3.2.2. Conversion between compartment-based and individual particle models

Without loss of generality assume that the compartment-based model is employed in [*a*, *I*_2_] and the Brownian-based model is employed in [*I*_1_, *b*] with the two models being simultaneously employed in the blending region [*I*_1_, *I*_2_]. Compartment-based dynamics are simulated with compartments of size *h* in [*a*, *I*_2_]. There are *n*_*c*_ = (*I*_1_ − *a*)/*h* compartments in the purely compartment-based region, [*a*, *I*_1_], and *n*_2_ = (*I*_2_ − *I*_1_)/*h* compartments in the overlap region, [*I*_1_, *I*_2_]. The number of particles in compartment *i* is, as before, labelled *C*_*i*_ for *i* = 1, …, *n*_*c*_ + *n*_2_. Brownian particles are simulated off-lattice with positions updated according to the discretized SDE (2.4) in [*I*_1_, *b*].

In each time interval of length Δ*t*_*b*_ assume, without loss of generality, that the compartment-based solution is updated first, followed by the Brownian-based dynamics. During the propagation of the compartment-based solution, it is likely that the numbers of particles in the compartments of the blending region have changed. Consequently, we need to alter the positions of Brownian particles in the blending region. If a particle jumps from compartment *i* to a neighbouring compartment *j* in the hybrid region, then we select a Brownian particle uniformly at random from among the particles which currently reside in compartment *i* and move it a distance ±*h* with the sign of the displacement corresponding to the direction of the compartment-based particle’s jump, i.e.

3.11 $$y_{k}=y_{k}\pm h,$$

where *k* indexes the randomly selected Brownian particle from compartment *i*.

If a particle in compartment *n*_*c*_ + 1 (the first compartment in the blending region) jumps leftwards out of the blending region (according to the compartment-based jump rates) and into the purely compartment-based region then a Brownian particle in the compartment *n*_*c*_ + 1 is selected uniformly at random and removed from the simulation (as well as particle numbers in the affected compartments being updated). Conversely, if a compartment-based particle jumps to the right, out of the last compartment in the purely compartment-based regime into the first compartment in the blending region, then a Brownian particle is added with its position chosen uniformly at random in this compartment, [*I*_1_, *I*_1_ + *h*] (as well as particle numbers in the affected compartments being updated). Note that the jump rates in the compartment-based model, which implement diffusion corresponding to equation (2.2), are such that, with our chosen blending diffusion coefficients, the rate of jumping to the right out of the final compartment is zero, so that no compartment-based particles can erroneously jump into the purely Brownian regime. Similarly, the diffusion coefficient of the Brownian particles at the pure-compartment/blending region interface is zero. Technically, with our finite time-step implementation of diffusion it might be possible for Brownian particles to erroneously jump over the interface into the purely compartment-based regime.^5^ On the rare occasions that a Brownian particle is chosen to jump over the interface (as an artefact of the numerical implementation) we simply reflect it back into the blending region. Since the diffusion coefficient is low in the boxes close to the interface this very rarely happens, and when it does the error caused by reflecting the particle is minimal.

Once the particle-based method has been propagated, it is usually necessary to update the number of particles in the compartments of the blending region, *C*_*i*_ for *i* = *n*_*c*_ + 1, …, *n*_*c*_ + *n*_2_. Rather than tracking every Brownian particle movement to see whether it has crossed over a compartment boundary, instead we simply sum the number of Brownian-based particles in each compartment at the end of the Brownian update to find the numbers of particles in each compartment of the blending region,

3.12 $$C_{n_{c}+i}=\sum_{k=1}^{N}I_{y_{k}\in [I_{1}+(i-1)h,I_{1}+ih]},\;\text{for}\;i=1,\ldots ,n_{2},$$

where $I_{y\in [I_{1}+(i-1)h,I_{1}+ih]}$ is the indicator function which takes the value 1 if the Brownian particle lies in the (*n*_*c*_ + *i*)th compartment and 0 otherwise.

Reactions in the blending region (similarly to the PDE–compartment hybrid method) are always implemented using the compartment-based paradigm. If a reaction occurs in the hybrid region then the appropriate Brownian particles are added (with positions chosen uniformly at random across the corresponding compartment) or removed (with the particle(s) selected uniformly at random from among those in the compartment).

### 3.3. Coupling algorithms

Having established the conversion rules in the previous section, we are now in the position to present two-hybrid algorithms. In particular, algorithm 1 is the algorithm that couples diffusion in the PDE and compartment-based models, while algorithm 2 is the algorithm that couples diffusion in the compartment-based models with Brownian-based dynamics. We have presented both of these algorithms with maximum generality in order to emphasize that the specific simulation methodologies are not important. In the next section, we implement these algorithms with a finite-volume PDE solver, the spatial Gillespie algorithm for compartment-based dynamics and the *λ* − *ρ* Brownian reaction–diffusion paradigm for the Brownian dynamics, in order to provide concrete examples of their implementation. Algorithms for the implementation of these three methods are given in appendices A, B and C, respectively.

**Algorithm 1.** Coupling a PDE solution with a compartment-based approach.

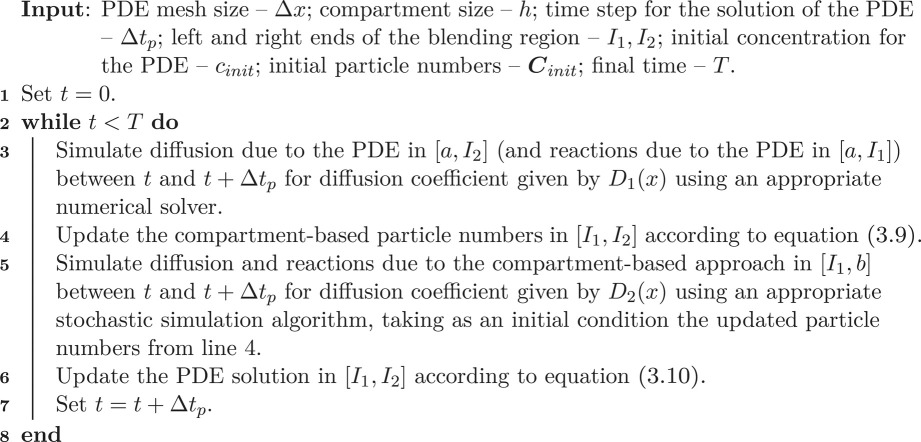

**Algorithm 2.** Coupling a compartment-based approach with Brownian dynamics.

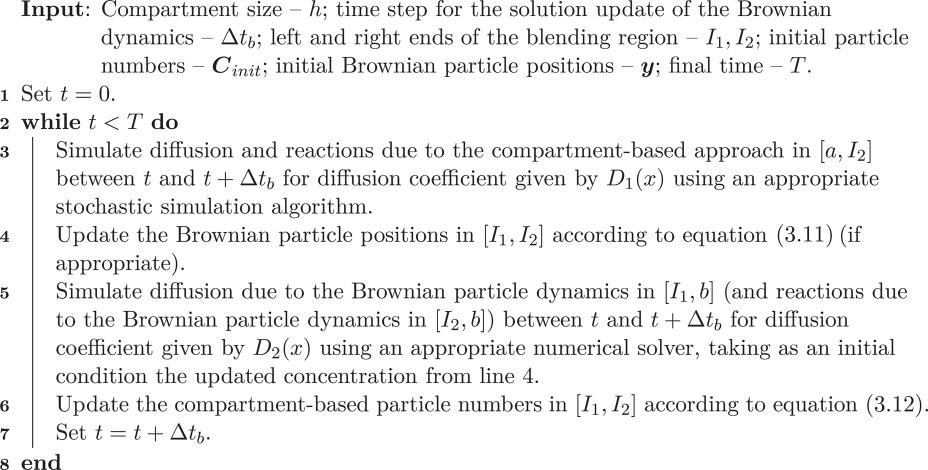

## 4. Results

In this section, we demonstrate that our proposed algorithms correctly simulate four test problems of increasing complexity. The first two problems are simulations of pure diffusion with different initial conditions, demonstrating that the fluxes over the interface of the hybrid model are consistent with the expected behaviour of the finer-scale representation in each hybrid model. The third problem, one of morphogen gradient formation, evidences the successful implementation of reactions in our hybrid algorithms. Finally, in the fourth test problem, we implement a second-order reaction system in three dimensions, demonstrating the applicability of the method to more complicated scenarios.

For each of the first three test problems, the one-dimensional domain we employ is *Ω* = [*a*, *b*] = [0, 1], with *I*_1_ = 1/3 and *I*_2_ = 2/3. The remainder of the parameter values for examples 1 and 2 are specified in table 1, for example 3 in table 2 and for example 4 in table 3. The blending functions for these three problems (and by simple extension for the fourth problem) are defined as the simple linear functions

4.1 $$f_{1}(x)=2-3x$$

and

4.2 $$f_{2}(x)=3x-1,$$

**Table 1.** Parameter values used for the pure diffusion simulation of test problems 1 and 2.

parameter	value	description
*N*	1000	number of particles
*Ω*	[0, 1]	spatial domain
*D*	1	diffusion coefficient
*K*	20	number of compartments
*h*	1/30	compartment width
Δ*x*	1/300	PDE voxel width
Δ*t*_*p*_	10^−4^	PDE time step
Δ*t*_*b*_	10^−4^	Brownian time step
*M*	500	number of repeats

**Table 2.** Parameters for the morphogen gradient simulation (test problem 3).

parameter	value	description
*N*(0)	1000	initial number of particles
*Ω*	[0, 1]	spatial domain
*D*	1	diffusion coefficient
*J*	10 000	rate of influx at the left boundary
*μ*	10	rate of particle decay
*K*	20	number of compartments
*h*	1/30	compartment width
Δ*x*	1/300	PDE voxel width
Δ*t*_*p*_	10^−4^	PDE time step
Δ*t*_*b*_	10^−4^	Brownian time step
*M*	1000	number of repeats

**Table 3.** Parameters for the bimolecular reaction simulation (4.10) (test problem 4).

parameter	value	description
*N*(0)	465	initial number of particles
*Ω*	[0, 10] × [0, 1] × [0, 1]	spatial domain
*D*	1	diffusion coefficient
*κ*_1_	0.1	rate of degradation reaction (see system (4.10))
*κ*_2_	89.7	rate of production reaction (see system (4.10))
*ρ*	0.06	particle interaction radius
Δ*t*_*b*_	10^−4^	Brownian time step
*P*_*λ*_	2.5 × 10^−5^	probability of reaction when inside the interaction radius
*K*	20	number of compartments
*h*_*x*_	1/3	compartment width
*h*_*y*_	1	compartment depth
*h*_*z*_	1	compartment height
Δ*x*	1/300	PDE voxel width
Δ*t*_*p*_	10^−4^	PDE time step
*M*	500	number of repeats

which scale the contribution of each method to the diffusion coefficient linearly between 0 and 1 across the blending region. These, in conjunction with equations (3.3) and (3.4), define the diffusion coefficients for both regimes across the whole domain. For each of the first three examples and for both couplings, we will quantify the qualitative comparisons (provided by density comparison snapshots) with error plots displaying the evolution of the difference between the averaged profiles of our hybrid methods and the mean-field PDE (see equations (4.3)–(4.6)). In the fourth example (for which the PDE is not an exact description of the mean behaviour of the individual-based methods), we will compare the averaged profiles of our hybrid methods with the averaged profiles of the finer-scale ‘ground truth’ (e.g. mesoscale or miscroscale) simulations (see equations (4.15)–(4.20)).

### 4.1. Test problem 1: uniform distribution

The first test of our hybrid algorithms is to determine whether, when simulating diffusion, they are capable of maintaining the uniform steady-state distribution across the domain without introducing any bias. We initialize particles uniformly across the domain and implement zero-flux boundary conditions.

**Figure 2.**
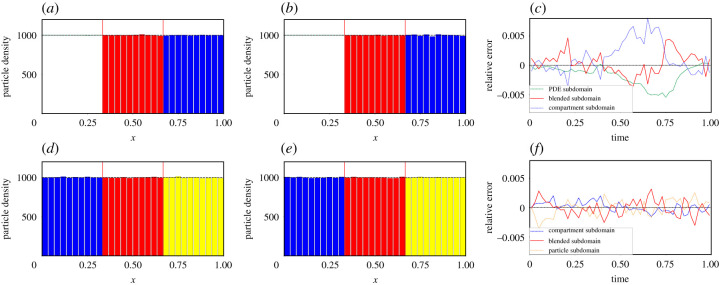
Density and error plots for test problem 1—pure diffusion with a uniform initial condition. (*a*–*c*) The PDE–compartment hybrid method and (*d*–*f*) the compartment–Brownian hybrid method. (*a*,*d*) Snapshots at time 0.1, and (*b*,*e*) at time 1. In (*a*,*b*), the green line is the PDE part of the hybrid method, the red bars represent the number of particles in each compartment in the blending region and the blue bars represent the number of particles in each compartment in the purely compartment-based region. In (*d*,*e*), the blue bars represent the number of particles in each compartment in the purely compartment-based region, the red bars represent the number of particles in each compartment in the blending region and the yellow bars the number of particles (appropriately binned for visualization purposes) in the purely Brownian region. In all four density comparison panels, the black dashed line represents the analytical solution of the diffusion equation with the given initial condition. Vertical red lines mark the position of the interfaces. (*c*) The relative error (described in the main text) between the density given by the PDE–compartment hybrid method and the density given by the analytical solution of the diffusion equation with the same initial condition. Similarly, (*f*) shows the relative error (described in the main text) between the density given by the compartment–Brownian hybrid method and the density given by the analytical solution of the diffusion equation with the same initial condition. Results shown are for *N* = 1000 particles and are averaged over 500 repeats. All other parameters are given within table 1.

**Figure 3.**
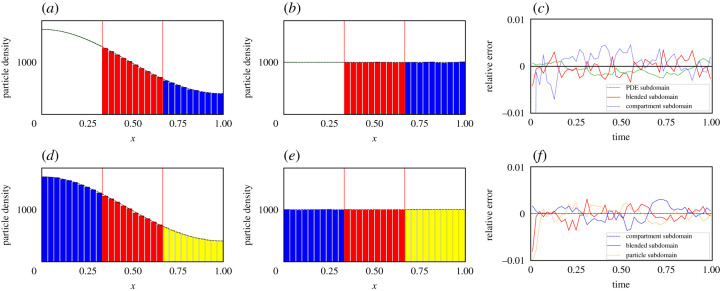
Density and error plots for test problem 2—pure diffusion with a step function initial condition in [*a*, *I*_1_]. Descriptions, including definitions of relative errors, are as in figure 2.

**Figure 4.**
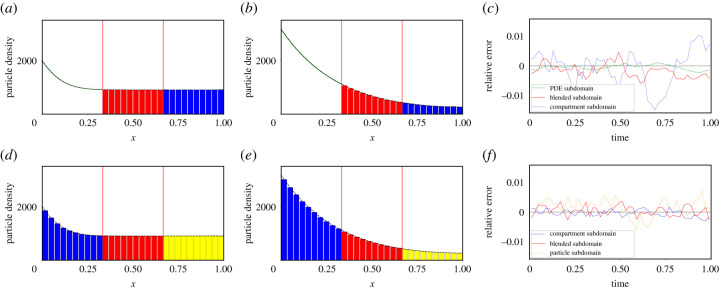
Density and error plots for test problem 3—morphogen gradient formation with a uniform initial condition. Descriptions, including definitions of relative errors, are as in figure 2 except that panels (*a*) and (*d*) are density profiles evaluated at *t* = 0.01 rather than at *t* = 0.1. All figure parameters are given in table 2.

In figure 2 (as well as for figures 3 and 4), the top three figures are for the PDE–compartment coupling and the bottom three figures for the compartment–Brownian coupling. The left-most panels display the density profile of the hybrid methods at time *t* = 0.1 and the middle panels the density profile at *t* = 1. In both left and middle panels, the mean behaviour of the stochastic model simulated across the whole of the domain is displayed as a black, dashed line for comparison. The right–most panels display the evolution through time of the relative mass error (RME) of each region of the domain: [*a*, *I*_1_], [*I*_1_, *I*_2_] and [*I*_2_, *b*]. For the PDE–compartment coupling, the RME is the difference between the average (over 500 repeats—unless otherwise stated) number of particles in the given region in the hybrid method and the corresponding number in the same region in the analytical solution of the PDE, *u*(*x*, *t*), divided by the number of particles in the relevant region of the analytical solution of the PDE (to normalize),

4.3 $${\text{RME}}_{P}(t)=\frac{\int_{\Omega_{P}}\;\bar{\;c}(x,t)\;\text{d}x-\int_{\Omega_{P}}u(x,t)\;\text{d}x}{\int_{\Omega_{P}}u(x,t)\;\text{d}x},$$

4.4 $${\text{RME}}_{H}(t)=\frac{\sum_{i}\;{\bar{\;C}}_{i}(t)I_{c_{i}\in \Omega_{H}}-\int_{\Omega_{H}}u(x,t)\;\text{d}x}{\int_{\Omega_{H}}u(x,t)\;\text{d}x}$$

4.5 $$\;\mathrm{and}\;\;\;{\text{RME}}_{C}(t)=\frac{\sum_{i}\;{\bar{\;C}}_{i}(t)I_{c_{i}\in \Omega_{C}}-\int_{\Omega_{C}}u(x,t)\;\text{d}x}{\int_{\Omega_{C}}u(x,t)\;\text{d}x},$$

where *Ω*_*P*_ = [*a*, *I*_1_] is the purely PDE region of the domain, *Ω*_*H*_ = [*I*_1_, *I*_2_] is the blending region and *Ω*_*C*_ = [*I*_2_, *b*] is the purely compartment region of the domain. The averaged solution of the PDE component of the hybrid method at position *x* at time *t* is denoted $\;\bar{\;c}(x,t)$ and the averaged compartment particle numbers in voxel *i* of the hybrid method are denoted $\;{\bar{\;C}}_{i}$. The positions *c*_*i*_ are the centres of the compartments.

For the compartment–Brownian coupling, the RME is the difference between the average (over 500 repeats—unless otherwise stated) number of particles in each region given by the hybrid method and the number of particles in the analytical solution of the mean-field PDE model in the corresponding region, divided by the number of particles in the relevant region of the analytical solution of the PDE (to normalize). In the pure compartment and blending regions, these are given by equations (4.5) and (4.4), respectively, with the altered definition of *Ω*_*C*_ = [*a*, *I*_1_] for equation (4.5). For the purely Brownian region, the RME is given by

4.6 $${\text{RME}}_{B}(t)=\frac{\;\bar{\;B}-\int_{\Omega_{B}}u(x,t)\;\text{d}x}{\int_{\Omega_{B}}u(x,t)\;\text{d}x},$$

where *Ω*_*B*_ = [*I*_2_, *b*] and $\;\bar{\;B}$ represents the average number of Brownian particles in the purely Brownian regime.

Figure 2 demonstrates that both of our hybrid blending methods pass this most basic test of maintaining a uniform distribution across the domain. The interfaces between the different modelling regimes are effectively undetectable. Qualitatively, the density plots all show good agreement between the hybrid methods and the analytical solution to the mean-field diffusion equation. This is confirmed by the relative error plots (figure 2*c*,*f*), which demonstrate low errors that fluctuate around zero with no discernible long-term bias.

### 4.2. Test problem 2: particle redistribution

The second test problem is designed to determine whether the hybrid methods can cope with high levels of flux across their interfaces. As with the previous example, we model pure diffusion with no reactions, but this time with a different initial condition. All the particles are distributed uniformly within [*a*, *I*_1_] and the system is allowed to equilibrate. The results of these simulations for both the PDE–compartment hybrid and the compartment–Brownian hybrid are given in figure 3.

In figure 3, we have initialized the particles uniformly in the left-hand-most third of the domain, corresponding to the purely PDE region in the PDE–compartment hybrid and the purely compartment-based region in the compartment–Brownian hybrid.^6^ As in test problem 1, both of our hybrid methods correctly match the evolution of the density of the mean-field diffusion equation, as evidenced quantitatively by the relative error plots 3*c* and *f* .

### 4.3. Test problem 3: a morphogen gradient formation model

The formation of a morphogen gradient from a uniform initial condition constitutes the third test of our hybrid simulation algorithms. Particles are allowed to diffuse freely throughout the domain and degrade at a rate *μ*. To counteract the degradation and ensure a non-trivial steady state, particles are introduced at the left-hand boundary, *x* = *a* = 0, with flux *DJ*, and a zero-flux boundary condition is implemented at *x* = *b* = 1. Since the reactions we have introduced are first order, the continuum mean-field model corresponding to the described set-up is governed by the following PDE:

4.7 $$\frac{\partial c}{\partial t}=D\frac{\partial^{2}c}{{\partial x}^{2}}-\mu c,\;\text{for}\;x\in (0,1)\;\text{and}\;t\in (0,T),$$

with boundary conditions

4.8 $$\frac{\partial c}{\partial x}(0,t)=-J,\;\frac{\partial c}{\partial x}(1,t)=0,\;t\in (0,T),$$

and initial condition

4.9 $$\;c(x,0)=c_{0},\;\text{for}\;x\in [0,1],\text{where}\;c_{0}=\frac{DJ}{\mu }.$$

The initial condition is chosen so that we begin with the same number of particles as there will be at steady state, but distributed uniformly across the domain.^7^ The parameters we employ for the simulations shown in figure 4 are given in table 2. Specifically, influx parameter, *J*, and degradation parameter, *μ*, are chosen to ensure an average of 1000 particles populating the domain throughout the simulation.

Figure 4 illustrates the solutions of our two-hybrid methods and those of the corresponding mean-field model (given by equation (4.7)). As with the previous two test problems, qualitative density profiles are in close agreement and quantitative error plots show low error and no sustained bias about zero.

### 4.4. Test problem 4: bimolecular production-degradation

The final scenario we will use to demonstrate the accuracy of our hybrid methods is a system of diffusing particles interacting through the following pair of chemical reactions:

4.10 $$2A\overset{\kappa_{1}}{\to }\emptyset ,\;\emptyset \overset{\kappa_{2}}{\to }A,$$

which occur within the cuboidal domain $\Omega \subseteq R^{3}$ of volume *V*, where *Ω* = [0, 10] × [0, 1] × [0, 1].

The blending hybrid method is extended to this three-dimensional example in the natural way. As in the one-dimensional test problems, the domain is divided into three equally sized subdomains, this time with planar interfaces, *I*_1_ at *x* = 10/3 and *I*_2_ at *x* = 20/3. The compartment-based region for each hybrid method is divided into a lattice of cuboidal compartments of size *h*_*x*_ × *h*_*y*_ × *h*_*z*_. The blending region is itself a cuboidal region in which both the coarse and fine models coexist as equivalent representations of the mass in that region. For this translationally invariant example, the blending functions are simply a function of *x*. This means that only diffusion parallel to the *x*-axis is impacted in the blending region. Of course, for differently shaped domains and interfaces, the blending functions may be functions of all three coordinates chosen to scale diffusion as required, providing *f*_1_(*x*, *y*, *z*) + *f*_2_(*x*, *y*, *z*) = *D* for all (x,y,z)∈B, the blending region, and both *f*_1_(***I_1_***) = *f*_2_(***I_2_***) = *D* and *f*_1_(***I_2_***) = *f*_2_(***I_1_***) = 0 are satisfied, where $I_{1}\in R^{3}$ and $I_{2}\in R^{3}$ are surfaces specifying the interfaces which form the boundaries of the blending region.

The mean-field PDE that corresponds to the reaction system (4.10) under the Poisson moment closure assumption is given by

4.11 $$\frac{\partial c}{\partial t}=D\nabla^{2}c-\kappa_{1}c^{2}+\kappa_{2},$$

with corresponding zero-flux boundary conditions on each of the domain’s boundaries

4.12 $${\frac{\partial c}{\partial n}|}_{\partial \Omega }=0.$$

For the simulations whose results are displayed in figure 6, we initialize the particles according to a linear gradient so that the initial density decreases in the positive *x*-direction. Explicitly particle density profiles are initialized according to the following density profile:

4.13 $$c(x,y,z)=\frac{183-18x}{2},\;\text{for}\;(x,y,z)\in [0,10]\times [0,1]\times [0,1],$$

giving *N* = 465 particles initially. The PDE part of the hybrid simulation can be initialized exactly according to this profile. For the regions of the domain modelled by stochastic components of the hybrid method (e.g. in compartment-based regions or Brownian-based regions) the density profile is normalized and used as a probability density function (PDF) to assign positions to the appropriate number of particles corresponding to that region of the domain. In the blending regions, particles are initialized according to the finer-scale simulation method and the coarse scale density is matched appropriately. For example, in the compartment–Brownian hybrid method, we initialize, on average, one-third of the particles with *y* and *z* coordinates chosen uniformly at random in [0, 1] and *x*-coordinates chosen from the PDF

4.14 $$P(x)=\left\{ \begin{array}{cc} 0\; & \text{for}\;0\leq x<\frac{10}{3},\;\\ \frac{183-18x}{310}\; & \text{for}\;\frac{10}{3}\leq x<\frac{20}{3},\;\\ 0\; & \text{for}\;\frac{20}{3}\leq x<10.\; \end{array}\right.$$

Once the positions of the Brownian particles have been specified, the particles can then be binned into compartments to determine the compartment-based initial condition in that region.

**Figure 5.**
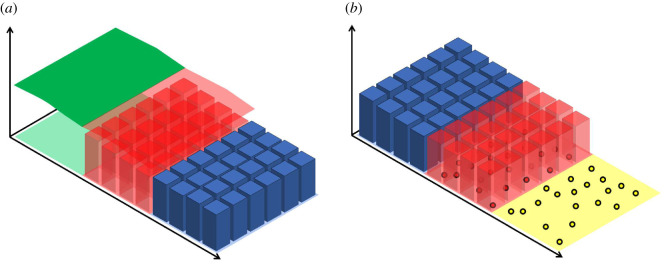
Schematic of the two-dimensional (*a*) PDE–compartment hybrid and (*b*) compartment–Brownian hybrid. In (*a*), the green surface in the green region represents the PDE solution in the purely PDE region of the domain. The red surface and the red columns represent equivalent PDE- and compartment-based representations of the mass in the red blending region. The blue columns in the blue region of the domain represent the number of particles in each compartment in the purely compartment region of the domain. In (*b*), the blue boxes in the blue region of the domain represent the number of particles in each compartment in the purely compartment region of the domain. The red boxes and the red circles represent equivalent compartment- and Brownian-based representations of the mass in the red blending region. The yellow circles in the yellow region of the domain represent individual particles in the purely Brownian region of the domain.

The hybrid method in three dimensions proceeds in an entirely analogous way to the one-dimensional algorithms described above with full three-dimensional simulation of the PDE, compartment-based method and Brownian-based method. As before, in the blending region the two different descriptions are kept in synchronization with each other at every time step. Figure 5 provides a schematic of the two coupling methods in two dimensions (in order to illustrate how the method generalizes from one dimension).

**Figure 6.**
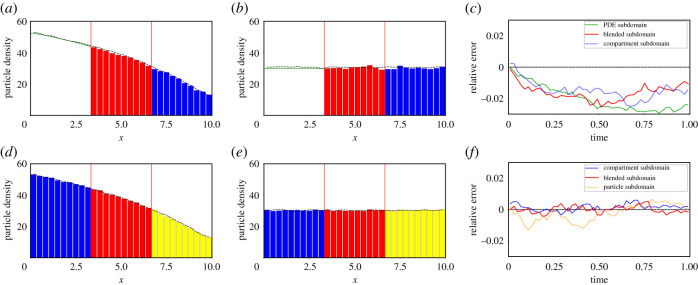
Density and error plots for test problem 4 with an initial condition which exhibits a constant gradient. Descriptions, excluding definitions of relative errors, are as in figure 2. All parameters are given in table 3.

**Figure 7.**
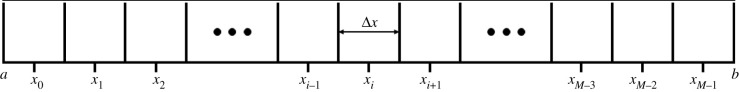
Schematic illustrating a spatial discretization of the one-dimensional domain [*a*, *b*], which is used to simulate equation (2.2) numerically. Δ*x* represents the size of the voxels and *x*_*i*_ for *i* = 0, …, *M* − 1 represent their centres.

The layout for figure 6 is the same as for figures 2–4. The only difference is the calculation of the RME. For this final example, which includes second-order reactions, the solution of the mean-field PDE model will no longer match the mean behaviour of either the compartment-based model or the Brownian-based model. Consequently, in order to calculate the RME, we use the average behaviour of the finest-scale model in each hybrid representation (e.g. the compartment-based representation in the PDE–compartment model and Brownian-based representation in the compartment–Brownian model) simulated across the whole domain as the ground truth. For the PDE–compartment coupling the RME is, then, the difference between the average (over 500 repeats) number of particles in the given region in the hybrid method and the corresponding average (over 500 repeats) number in the same region in the purely compartment-based simulation, divided by the number of particles in the relevant region of the purely compartment-based simulation (to normalize)

4.15 $${\text{RME}}_{P}(t)=\frac{\int_{\Omega_{P}}\;\bar{\;c}(x,y,z,t)\;\text{d}x-\sum_{i,j,k}{\bar{F}}_{i,j,k}(t)I_{c_{i,j,k}\in \Omega_{P}}}{\sum_{i,j,k}{\bar{F}}_{i,j,k}(t)I_{c_{i,j,k}\in \Omega_{P}}},$$

4.16 $${\text{RME}}_{H}(t)=\frac{\sum_{i,j,k}\;{\bar{\;C}}_{i,j,k}(t)I_{c_{i,j,k}\in \Omega_{H}}-\sum_{i,j,k}{\bar{F}}_{i,j,k}(t)I_{c_{i,j,k}\in \Omega_{H}}}{\sum_{i,j,k}{\bar{F}}_{i,j,k}(t)I_{c_{i,j,k}\in \Omega_{H}}}$$

4.17 $$\;\mathrm{and}\;{\text{RME}}_{C}(t)=\frac{\sum_{i,j,k}\;{\bar{\;C}}_{i,j,k}(t)I_{c_{i,j,k}\in \Omega_{C}}-\sum_{i,j,k}{\bar{F}}_{i,j,k}(t)I_{c_{i,j,k}\in \Omega_{C}}}{\sum_{i,j,k}{\bar{F}}_{i,j,k}(t)I_{c_{i,j,k}\in \Omega_{C}}},$$

where, as before, *Ω*_*P*_ is the purely PDE region of the domain, *Ω*_*H*_ is the blending region and *Ω*_*C*_ is the purely compartment region of the domain. The averaged solution of the PDE component of the hybrid method at position (*x*, *y*, *z*) at time *t* is denoted $\;\bar{\;c}(x,y,z,t)$, the averaged compartment particle numbers in compartment (*i*, *j*, *k*) of the hybrid method are denoted $\;{\bar{\;C}}_{i,j,k}$ and the averaged compartment particle numbers in compartment (*i*, *j*, *k*) of the fully compartment-based ‘ground truth’ simulation are denoted ${\bar{F}}_{i,j,k}$. The positions *c*_*i*,*j*,*k*_ are the centres of the compartments indexed (*i*, *j*, *k*).

For the compartment–Brownian coupling, the RME is the difference between the average (over 500 repeats) number of particles in each region given by the hybrid method and the average number of particles in the same region in the purely Brownian-based simulation, divided by the number of particles in the relevant region of the purely Brownian-based simulation (to normalize):

4.18 $${\text{RME}}_{C}(t)=\frac{\sum_{i,j,k}\;{\bar{\;C}}_{i,j,k}(t)I_{c_{i,j,k}\in \Omega_{H}}-{\bar{E}}_{C}(t)}{{\bar{E}}_{C}(t)},$$

4.19 $${\text{RME}}_{H}(t)=\frac{\sum_{i,j,k}\;{\bar{\;C}}_{i,j,k}(t)I_{c_{i,j,k}\in \Omega_{H}}-{\bar{E}}_{H}(t)}{{\bar{E}}_{H}(t)}$$

4.20 $$\;\mathrm{and}\;{\text{RME}}_{B}(t)=\frac{\;\bar{\;B}(t)-{\bar{E}}_{B}(t)}{{\bar{E}}_{B}(t)},$$

where, as before, *Ω*_*B*_ is the purely Brownian region of the domain, $\;\bar{\;B}(t)$ represents the mean number of Brownian particles in the purely Brownian region of the hybrid method and ${\bar{E}}_{C}(t)$, ${\bar{E}}_{H}(t)$ and ${\bar{E}}_{B}(t)$ represent the mean number of Brownian particles in *Ω*_*C*_, Ω_*H*_ and *Ω*_*B*_, respectively, at time *t* in the fully Brownian ‘ground truth’ simulations.

There are some special points to note about the models which incorporate second-order reactions. Firstly, as noted above, the solution of mean-field PDE, which we will employ in the PDE region of the PDE–compartment hybrid method, will not correspond to the mean behaviour of the compartment-based method. This is a result of the moment-closure approximation, which must be used in order to derive a closed PDE for the mean behaviour. As a consequence, we might expect some disparity between the solution of the hybrid method and the solution of the fully compartment-based simulation that we take to be the ground truth in the PDE–compartment-based hybrid. Fortunately, for our compartment–Brownian hybrid method, Erban & Chapman [51] provide a method for matching reaction rates in compartment-based simulations to those in Brownian-based simulations, which we make use of.

We must also be careful to choose our parameters carefully in the compartment–Brownian method. If compartment sizes are too small in the compartment-based method then particles can become too sparsely distributed and second-order reactions lost. Erban & Chapman [51] provide a way to alter the reaction rate (depending on the compartment size) to maintain the same overall reaction rate as a well-mixed system. This correction, however, only holds down to a certain compartment size, beyond which second-order reactions are irrevocably lost. It is worth noting that Isaacson [46] postulated the convergent reaction–diffusion master equation representation (in which particles can interact with others in neighbouring boxes), which is consistent with the spatially continuous Doi model for reaction–diffusion even as box sizes become small. Hellander *et al.* [69] numerically approximate mesoscopic reaction rates that are consistent with the popular Smoluchowski Brownian dynamics model up to a given lower limit on mesh size.

In the Brownian-based method, we need to ensure that the time step is chosen to be sufficiently small that particles do not jump ‘too far’ between position updates. If particles jump large distances in each time step then it is possible that particles which should have been given the opportunity to react with each other may not come into close enough proximity and some second-order reactions may be lost. Choosing the reaction radius of particles to be large may help to mitigate this somewhat, but brings its own problems. The size of the interaction radius is calculated by considering particles in free space [51]. In reality, in our simulations, particles are often close to boundaries. The proportion of the particle’s interaction radius that overlaps the exterior of the domain is not able to interact with particles inside the domain, so the rate of second-order reactions is again effectively reduced. Since, for a given reaction rate, the size of the interaction radius increases with the time step, reducing the time step is often sufficient to solve both of these problems. We note that, with the exception of the PDE not matching the mean behaviour of the compartment-based method, these issues are all inherent to the individual modelling paradigms we have chosen to couple, and are not specific to the hybrid methods we have developed. With sensible simulation parameter choices these issues can be overcome.

The results of our simulations are plotted in figure 6. In figure 6*d*,*e*, which compares densities for the compartment–Brownian hybrid paradigm, we have good qualitative agreement with the ground truth (the ubiquitously Brownian-based model). These qualitative results are further corroborated in figure 6*f*, in which the low and unbiased RME over time are demonstrated.

The density plots in figure 6*a*,*b* for the PDE–compartment hybrid coupling also appear to demonstrate good qualitative agreement. However, when considering the RME in the different regions, in figure 6*c*, we observe that, although low, the RMEs appear to be biased. This, as discussed above, should not be a surprise since the mean-field PDE does not capture the mean behaviour of the compartment-based model, which we assume to be the ground truth for the RME calculations. The overall mass expected in the fully compartment-based model at equilibrium would exceed that predicted by the mean-field PDE. In agreement with this expectation, we find that the total mass in all three regions of the domain is less than it would be in the fully compartment-based simulations with the problem being particularly acute in the PDE region. A simple comparison of the expected densities at time *t* = 1 shows that the maximum magnitude of the PDE relative error with respect to the compartment-based model is roughly 3 × 10^−2^, demonstrating that the size of the relative error we find between our hybrid method and the solution of the fully compartment-based simulations is of an appropriate order or magnitude, as it is similar to the difference in the concentration when comparing the equilibrium profile of the full PDE to the fully compartment-based method, adjusted for the specific voxel size.

## 5. Discussion

When modelling multiscale phenomena it is often the case that concentrations vary spatially to such a degree that in one region of the domain a coarse, computationally inexpensive model can be tolerated, whereas in another region of the domain a more accurate, but more expensive representation is required.

In this paper, we have proposed a general hybrid blending mechanism which facilitates the spatial coupling of two reaction–diffusion modelling paradigms at different levels of detail in order to accommodate the modelling of such multiscale phenomena. Our method employs a blending region and a corresponding blending function. The blending function scales up or down (respectively) the relative contribution to diffusion of a coarse or fine (respectively) representation of the reaction–diffusion process across the blending region such that diffusion is handled to a different degree by each modelling representation.

Specifically, we have developed an algorithm which couples a PDE representation of a reaction–diffusion process to a compartment-based representation and, separately, an algorithm which couples a compartment-based representation at the coarse scale to a Brownian-based representation at the fine scale. Other algorithms exist to achieve such couplings [12,14,16,33]. Some of these algorithms are conceptually complex—relying variously on artificially introduced ‘psuedo-compartments’, ‘ghost cells’ and ‘overlap regions’—technically challenging to implement and strongly parameter dependent—working only in specific parameter regimes. We believe our blending method provides a conceptually simple and easily implementable coupling methodology—requiring only an intuitively defined blending function to couple the two regimes together. This methodology might be readily employed to couple other modelling regimes (for example, PDE and Brownian modelling regimes) to form novel hybrid methods under a unified framework or implemented simply by non-experts for physical and biological applications.

We have demonstrated, through four representative examples, that both of our coupling algorithms are able to handle a wide range of reaction–diffusion processes from simple diffusion through to reaction–diffusion processes incorporating first- and second-order reactions. The hybrid methods are capable of representing these processes accurately (low error) and without bias (in the situation for which there is no discrepancy between mean-field behaviour of the coupled models) or with the expected bias (when such a discrepancy exists). Owing to the computational savings afforded by coupling a cheap coarse model with an expensive fine-scale model, we can scale up particle numbers in our simulations in order to demonstrate that the hybrid algorithms perform arbitrarily well in comparison to the full finest-scale model. For this reason, we do not provide explicit time comparisons of our methods, but rather focus on their accuracy.

There are several directions in which we intend to extend this work, but which are not appropriate for inclusion in this initial proof-of-principle paper. Firstly, and perhaps most straight-forwardly, we would like to extend these hybrid methods to deal with more complex domain geometries. Although we have demonstrated that our blending hybrid methods can cope with three-dimensional reaction–diffusion processes, in real biological scenarios boundaries are likely to be curved and there is the potential for the requirement that interfaces between coarse and fine regimes are non-planar.

Secondly, the dynamic nature of many biological processes mean that concentrations change significantly over time. If we are to ensure that the coarse modelling regime represents regions of high concentration and the fine modelling regime regions of low concentration, then it is necessary for interfaces that border the blending region and the blending region itself to be dynamic. The main challenge associated with dynamic interfaces is the conversion of one particle type into another. Fortunately, this challenge has been overcome previously by a number of different hybrid methods, whose dynamic interface methodologies we might readily adapt to our hybrid paradigm in follow-up work [19,26,30]. Related concerns are the need for the creation or removal of multiple interfaces in scenarios in which particle concentrations oscillate in space and time. Similarly, reaction–diffusion simulations in which more than just a single species are interacting may require different interfaces for each of the different species. This raises potentially difficult questions about how to carry out reactions between species represented by distinct modelling paradigms in the same region of space.

A final direction in which we would like to extend this work is by considering entirely new hybridization methods. For example, rather than having the two distinct modelling paradigms representing the same particles (as we have in the blending region), requiring both regimes to be updated when one changes, it might be practicable to have the two modelling paradigms coexisting across the whole of the domain, but representing different proportions of the particles depending on the concentration. Such a method would remove the requirement for interfaces between the regions of the domain, effectively doing away with many of the concerns related to dynamically and spatially changing concentrations raised earlier in this section.

Since biological and physical experiments can be carried out at increasingly high levels of detail, we are gaining more intricate and specific information about a wide variety of multiscale processes. In order to test experimentally generated hypotheses about such processes, we need to have modelling frameworks which are capable of replicating experimental behaviour to a high degree of accuracy. The blending hybrid methods presented in this paper provide a straightforward way to couple modelling paradigms with different levels of detail, which will facilitate more accurate and more efficient multiscale modelling. Consequently, we expect that both our own future work and the work of others, building on just such hybrid paradigms, will enable biochemical simulations which go beyond what is tractable with current approaches.

## Figures and Tables

**Figure 8.**
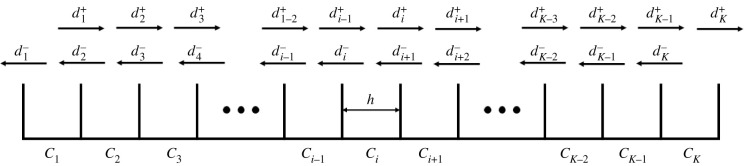
Schematic illustrating the jump rates, $d_{i}^{\pm }$, between compartments when simulating the mesoscopic reaction–diffusion paradigm on the domain [*a*, *b*] with compartment size *h*.

## Endnotes

1 Note that by a careful partitioning of space the number of comparisons can be reduced dramatically to almost *O*(*N*) when particles are only compared with others in their local neighbourhood [56].

2 As previously noted some of this complexity can be offset by a careful partitioning of space, allowing particles to be compared only with others in their local neighbourhood [56].

3 Note that, because of the choice of blending functions, the boundary conditions at *I*_2_ in (3.7a) and at *I*_1_ in (3.7b) are automatically satisfied.

4 Note that we describe the coupling between the two regimes in the blending region using the terminology of the finite-volume PDE discretization that we employ in our numerical examples (see §4). However, we also note that finite-volume voxels can be substituted for finite-difference or finite-element mesh points in a straightforward manner.

5 While there do exist integrators for diffusion processes which can guarantee that this situation does not happen [68], implementing such an approach is beyond the scope of the article.

6 We see similarly agreeable results when particles are initialized in the third of the domain [*I*_2_, *b*] corresponding to the purely compartment or purely Brownian regions, respectively.

7 Note that we have chosen this initial condition to ensure that the PDE–compartment algorithm functions appropriately. While the compartment-to-Brownian algorithm can deal naturally with low particle numbers, as noted earlier, there is the potential for low particle numbers to break the PDE–compartment algorithm. Potentially, when particle numbers are low in the blending region, fractional particle numbers in a compartment could cause a particle to be chosen to jump out of one compartment even though there is not sufficient mass for this to occur. The solution to this problem, as will be proposed in the Discussion, is to introduce adaptive blending regimes, which ensure that the PDE representation is only employed in regions of the domain where particle concentrations are sufficiently high to justify its use.

## Data accessibility

Code and some simulations from this paper are made available as the electronic supplementary material.

## Appendix A. Numerical simulation of the probability density function

We now provide more details on the specifics of the macroscopic model that we employ throughout the paper, including an algorithm for its implementation. There exist a number of well-developed, efficient numerical methods for the solution of such reaction–diffusion PDEs [70–73]. Typically to implement these algorithms, we discretize the PDE on a spatial mesh. This results in a system of ordinary differential equations (ODEs). These ODEs can then be integrated forwards in time using standard numerical techniques.

For the PDE (2.2), we start by dividing [*a*, *b*] into *M* voxels each of size Δ*x* = (*b* − *a*)/*M* and we define *x*_*j*_ = Δ*x*(*j* − 1/2), so that *x*_*j*_ is the centre of the voxel *j* (figure 7).Typically, the grid spacing of the PDE solution method is very fine (much finer than the discretization of space in the compartment-based method) in order to mimic the true continuous-space PDE solution as closely as possible. We discretize the PDE using the finite-volume method over the grid in figure 7. For time integration, we use the simple *θ*-method [72].

Below we provide a detailed implementation algorithm for the finite-volume PDE simulation method which is designed to replace line 3 in algorithm 1. We start by introducing

hboxA1 $$q_{\;j}(t)=\frac{1}{\Delta x}\int_{x_{\;j-1/2}}^{x_{\;j+1/2}}c(t,x)\;\text{d}x,$$

which corresponds to the average concentration per voxel, and *D*_*j*_ = *D*(*x*_*j*_), where we note that *j* is not necessarily integer valued. By integration of PDE (2.2) over the finite-volume voxels, we then obtain the semi-discrete approximation,

hboxA2 $$\frac{\text{d}q}{\text{d}t}=Aq+b+R(q),$$

where

$$A=\frac{1}{\Delta x^{2}}\left(\begin{array}{ccccc} -D_{1/2} & D_{1/2} & & & \\ D_{1/2} & -(D_{1/2}+D_{3/2}) & \;\;D_{3/2} & & \\ & \ddots & \ddots & \ddots & \\ & D_{M-5/2} & -(D_{M-5/2}+D_{M-3/2}) & D_{M-3/2}\\ & & D_{M-3/2} & -D_{M-3/2} \end{array}\right),$$

***b*** = Δ*x*^−1^( − *J*_*a*_, 0, …, 0, *J*_*b*_) and $q(t)=(q_{0}(t),q_{1}(t),\ldots ,q_{M-1}^{T}(t))$. We now solve the semi-discrete approximation using the *θ*-method.^8^ The complete method is described in algorithm 3.

**Algorithm 3.** An algorithm for the numerical solution of equation (2.2) using a first-order finite-volume method.

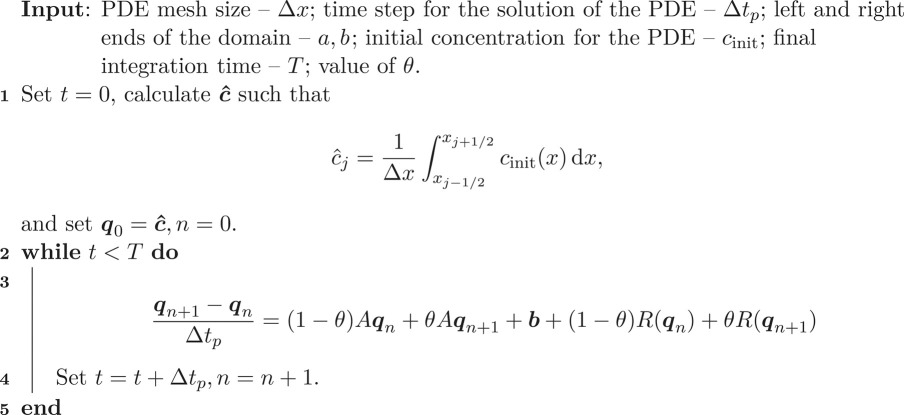

## Appendix B. Simulation of the compartment-based method

We now provide more details on the specifics of the mesoscopic model that we employ throughout the paper, including an algorithm for its implementation. More precisely, we have used an event-driven approach in order to simulate our compartment-based dynamics. The most commonly used event-driven algorithm for simulating Markov processes is the Gillespie direct method [74]. Each event is characterized by a propensity function which specifies the rate parameter of the exponentially distributed waiting time until the next ‘firing’ of that event. It can be shown that the time until the next reaction of any type (i.e. the minimum waiting time) is also exponentially distributed with a rate which is the sum of the rates of the individual reactions. Gillespie’s algorithm first generates an exponentially distributed minimum waiting time and subsequently, with probabilities proportional to their propensity functions, chooses a reaction to fire. Alternatively, time-driven algorithms can be employed, in which a sufficiently small time step is chosen such that the probability of more than one reaction/movement event firing in that time interval is negligible. Time-driven algorithms tend to be inefficient owing to the small time step required during which, typically, no change to the state is implemented. Consequently, exact event-driven algorithms tend to be favoured for the simulation of compartment-based dynamics.

Here, we discuss the implementation of a compartment-based reaction–diffusion model in one dimension with a spatially varying diffusion coefficient. Although we present our algorithm in one dimension it is straightforward to extend it to higher dimensions with planar interfaces. We give one such three-dimensional example in §4.4.

We first discretize the region [*a*, *b*] into *K* compartments, each of size *h* = (*b* − *a*)/*K*. In order to replicate the density-dependent diffusion specified in the macroscopic model described by equation (2.2), we require that the rates at which particles jump to the left and the right are not equal in regions in which the diffusion coefficient is non-constant. Specifically, we must evaluate the jump rates based on the diffusion coefficient at compartment boundaries [75]. This is visualized in figure 8, where we denote the jumping rate of a particle in compartment *i* into compartment *i* + 1 with $d_{i}^{+}$, while we denote the jumping rate of a compartment *i* particle into compartment *i* − 1 with $d_{i}^{-}$. Without loss of generality, assuming that the left-hand boundary of the compartment-based regime is at *a*, the left jump rate for compartment *i* is given by

hboxB1 $$d_{i}^{-}=\frac{D(a+(i-1)h)}{h^{2}},\;\text{for}i=2,\ldots ,K,$$

and the right jump rate is given by

hboxB2 $$d_{i}^{+}=\frac{D(a+ih)}{h^{2}},\;\text{for}\;i=1,\ldots ,K-1.$$

Jump rates $d_{1}^{-}$ and $d_{K}^{+}$ at the boundaries can be chosen in order to replicate the chosen boundary conditions [76]. In the case of zero-flux boundary conditions (equivalent to setting *J*_*a*_ = *J*_*b*_ = 0 in equations (2.3)), these jump rates are simply chosen to be $d_{1}^{-}=d_{K}^{+}=0$. Reaction propensity functions are specified to bring about the desired reaction rate.^9^ Once all the event rates have been specified then one can simulate the system using Gillespie’s direct method [74].

We next provide a detailed implementation for the spatial Gillespie algorithm over a time interval of size Δ*t*. This algorithm is designed to replace line 5 of algorithm 1 and line 3 of algorithm 2. Without loss of generality assume the compartment-based region occupies [*a*, *I*_2_], as in the compartment–Brownian hybrid method. However, we note the caveat that for the PDE–compartment hybrid method the compartment-based region would occupy [*I*_1_, *b*]. As already noted, left and right jumping rates from compartment *i* are different and given in equations (B 1) and (B 2), respectively.

**Algorithm 4.** Simulating the spatial Gillespie algorithm for a time interval Δt.

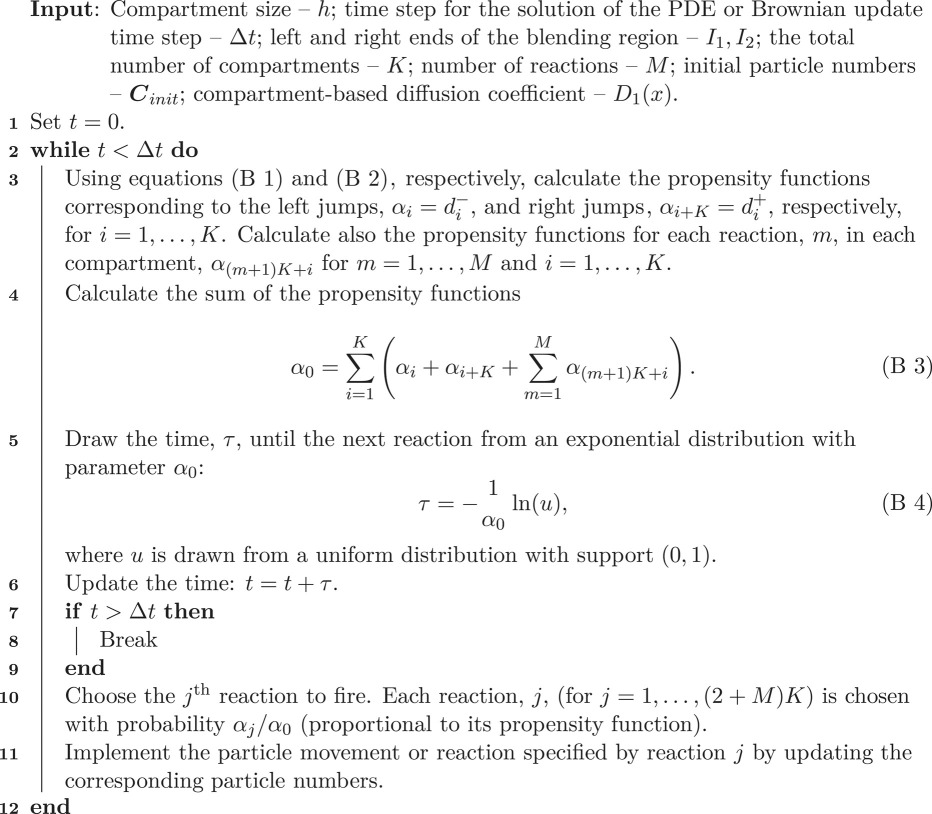

Note that the Gillespie algorithm steps forwards in discrete time steps. However, the time steps themselves are drawn from a continuous distribution so that the solution time points of the Gillespie algorithm do not match up with those of the fixed time-step algorithms for PDE and Brownian-based simulation. Consequently, our technique to couple the two simulation methodologies is to simulate the compartment-based dynamics until such a time as Δ*t* is exceeded for the first time. Since a PDE or Brownian update step is due at time Δ*t* we do not implement the final Gillespie reaction whose time step took us over the Δ*t* time limit. Instead, we implement a PDE or Brownian update step accordingly and correspondingly update the propensity functions ready to begin algorithm 4 again.

## Appendix C. Simulation of Brownian dynamics

In this appendix, we provide a provide a detailed implementation algorithm for Brownian-based dynamics, which is designed to replace line 5 in algorithm 2.

**Algorithm 5.** Simulating a Brownian update step of length $\Delta t_{b}$.

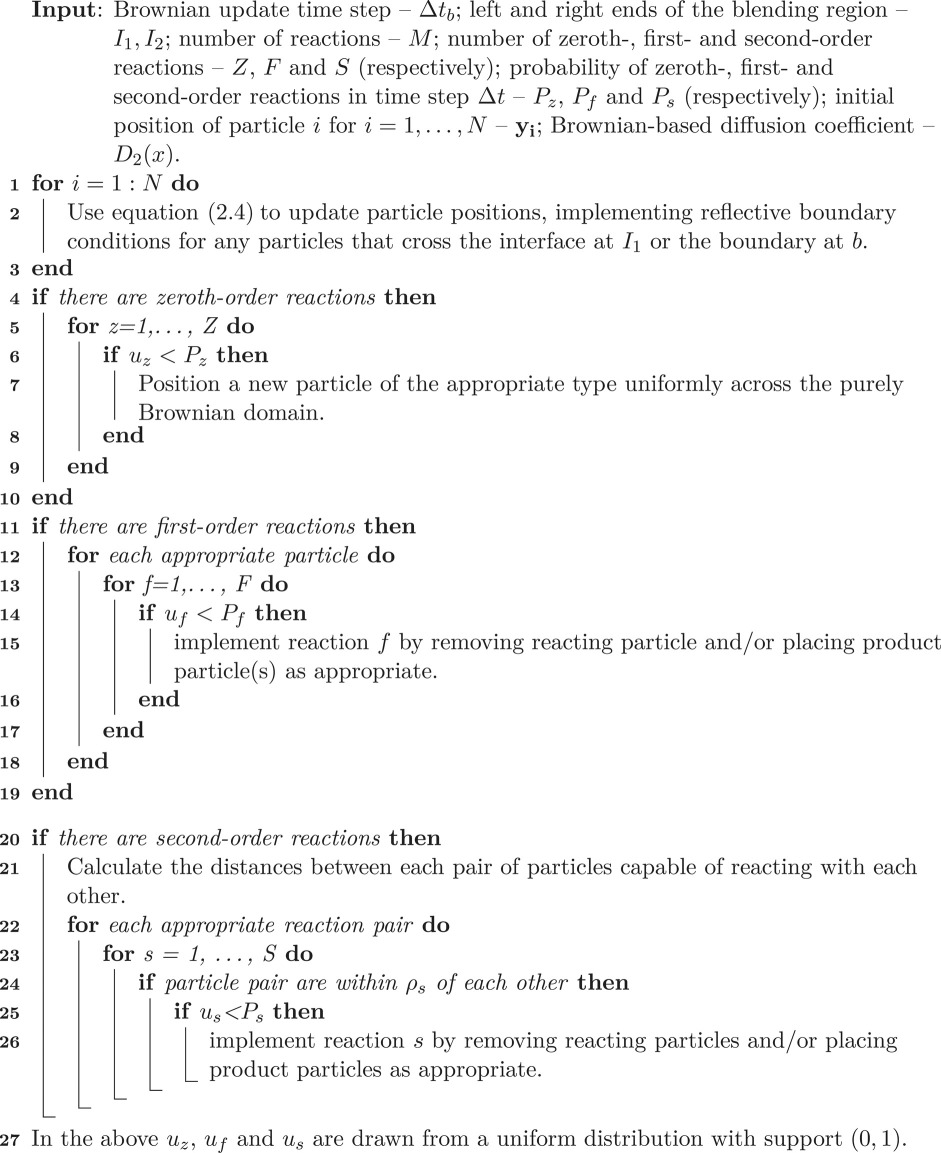

For zeroth- and first-order reactions, respectively, reaction probabilities, *P*_*z*_ and *P*_*f*_, respectively, are calculated simply by multiplying the rate of reaction by the time step, Δ*t*_*b*_. The calculation of *P*_*s*_ for second-order reaction, *s*, is somewhat more complicated and depends on the choice of reaction radius, *ρ*_*s*_. For more details on this and the placement of new particles after reaction, see [51]. Note that Brownian reactions are only implemented in the region [*I*_2_, *b*], since outside this region reactions are implemented using the compartment-based regime.

In theory, the fact that the diffusion coefficient of the Brownian-based particles falls to zero at *I*_1_ should mean that particles cannot cross the interface there, rendering the implementation of reflecting boundary conditions at *I*_1_ in step 2 redundant. However, in practice, the finite time step we use to update the Brownian particles means that, with low probability, particles can jump across the interface and must consequently be reflected back.
